# Diagnostic Accuracy of Machine Learning Models to Identify Congenital Heart Disease: A Meta-Analysis

**DOI:** 10.3389/frai.2021.708365

**Published:** 2021-07-08

**Authors:** Zahra Hoodbhoy, Uswa Jiwani, Saima Sattar, Rehana Salam, Babar Hasan, Jai K. Das

**Affiliations:** Department of Pediatrics and Child Health at the Aga Khan University, Karachi, Pakistan

**Keywords:** congenital heart disease, machine learning, diagnostic accuracy, meta-analysis, risk of bias

## Abstract

**Background:** With the dearth of trained care providers to diagnose congenital heart disease (CHD) and a surge in machine learning (ML) models, this review aims to estimate the diagnostic accuracy of such models for detecting CHD.

**Methods:** A comprehensive literature search in the PubMed, CINAHL, Wiley Cochrane Library, and Web of Science databases was performed. Studies that reported the diagnostic ability of ML for the detection of CHD compared to the reference standard were included. Risk of bias assessment was performed using Quality Assessment for Diagnostic Accuracy Studies-2 tool. The sensitivity and specificity results from the studies were used to generate the hierarchical Summary ROC (HSROC) curve.

**Results:** We included 16 studies (1217 participants) that used ML algorithm to diagnose CHD. Neural networks were used in seven studies with overall sensitivity of 90.9% (95% CI 85.2–94.5%) and specificity was 92.7% (95% CI 86.4–96.2%). Other ML models included ensemble methods, deep learning and clustering techniques but did not have sufficient number of studies for a meta-analysis. Majority (*n*=11, 69%) of studies had a high risk of patient selection bias, unclear bias on index test (*n*=9, 56%) and flow and timing (*n*=12, 75%) while low risk of bias was reported for the reference standard (*n*=10, 62%).

**Conclusion:** ML models such as neural networks have the potential to diagnose CHD accurately without the need for trained personnel. The heterogeneity of the diagnostic modalities used to train these models and the heterogeneity of the CHD diagnoses included between the studies is a major limitation.

## Introduction

The global prevalence of congenital heart disease (CHD) is six to nine children per 1,000 live births (Marelli et al., 2007; van der Linde et al., 2011). Although mortality due to CHD has halved in high income countries (HICs), low and middle income countries (LMICs) have seen a rise in disability and death in the last 20 years (IHME, 2015). Scaling up surgical care in these countries can reduce CHD related deaths by 58% (Higashi et al., 2015). However, prompt identification of patients is crucial to ensuring improved outcomes.

In HICs, the vast majority of children with CHD are diagnosed timely, mainly due to comprehensive pre- and postnatal screening (Lytzen et al., 2018). Echocardiography is considered to be the gold standard for diagnosis of pediatric and adult CHD (Mcleod et al., 2018). However, this diagnostic modality requires the existence of a healthcare system with appropriately trained personnel (Mcleod et al., 2018). The paucity of healthcare professionals in resource constrained areas means that many patients may depend on lesser trained health care providers for healthcare (Abdullah et al., 2014), resulting in higher rates of missed diagnoses and subsequent delays in treatment. Additionally, unavailability of echocardiographic machines, technologists, or expert interpretation in these areas may require many patients to travel large distances to tertiary care centers for confirmation of diagnosis.

Use of artificial intelligence (AI) in healthcare and its utility in medicine, from diagnosis and risk assessment to outcome predictions for a wide variety of illnesses has been extensively described in the literature (Koivu et al., 2018; Senders et al., 2018; Harris et al., 2019). The current developments in machine learning (ML), a subset of AI, has renewed the interest in using intelligent systems in healthcare. ML uses algorithms to allow computers to find patterns in data and make predictions without being given specific instructions (Beam and Kohane, 2018). The technology can analyze large amounts of complex data and identify previously unknown relationships. ML models are broadly classified as supervised, unsupervized, and semi-supervized when the data are fully labeled, unlabeled or partially labeled, respectively (Zhang, 2010). For an ML model to be successful and generalizable to new cases, the data from which it learns needs to be robust and sufficiently vast (Halevy et al., 2009).

The utility of ML in aiding diagnosis is not only beneficial in resource-limited areas, but presents universal opportunities for healthcare (Beam and Kohane, 2018). Specifically in cardiology, ML has potential applications in cardiac diagnostic imaging (Gandhi et al., 2018), electrocardiogram (ECG) interpretation (Mincholé et al., 2019), and auscultation (Leng et al., 2015), and therefore, has the potential to be used as a diagnostic aid for identification of structurally abnormal hearts and specific types of CHDs. The advances in AI in recent years have shown great improvements in recognition of cardiac shape, size and structure, thus presenting a potential solution to the scarcity of diagnostic services in LMICs. However, for ML to be fully incorporated in clinical care as a diagnostic tool, the accuracy of its diagnostic ability needs to be evaluated. The objective of this review is to estimate the diagnostic accuracy of ML models for detecting CHD diagnosed by an expert clinician or through echocardiography (reference standard).

## Materials and Methods

### Literature Search

The protocol for the review was prospectively registered at PROSPERO (CRD42020186672). A comprehensive literature search in the PubMed, CINAHL, Wiley Cochrane Library, and Web of Science electronic databases was performed to identify relevant articles published until March 31, 2020. The search strategy was (“Artificial Intelligence [Mesh]” OR “Artificial intelligence” OR “AI” OR (((“Machine”) OR (“Deep”) OR (“Ensemble machine”)) AND (“Learning”)) OR “Processing” OR (((“Supervised”) OR (“unsupervised”)) AND “learning”)) OR “Neural network*”) AND (“Heart Defects, Congenital [Mesh]” OR (((“ventric*”) OR (Atri*) OR (sept*)) AND (“defect”)) OR “tetralogy” OR (((“pulmonary”) OR (“tricuspid”)) AND (“atresia”)) OR “patent ductus” OR “transposition” OR (((“pulmonary”) OR (“aortic”)) AND (“stenosis”)) OR “Ebstein anomaly” OR “coarctation of aorta” OR “hypoplastic left heart” OR “truncus arteriosus”). All records were imported to Endnote X9 for management and duplicate records were deleted. Two authors (ZH and UJ) independently screened titles and abstracts to assess for potential eligibility. Full texts of all screened studies were reviewed for final selection. Titles of excluded literature along with the reason for exclusion were recorded. We followed the Preferred Reporting Items for Systematic reviews and Meta-Analyses (PRISMA) guidelines for diagnostic test accuracy for analysis reporting in this publication (McInnes et al., 2018).

### Eligibility Criteria

All cross-sectional, case-control and cohort studies that reported the diagnostic results of a ML algorithm for the detection of CHD as compared to a reference standard (categorized as an imaging and/or expert confirmation) and were published in English language were included. No restrictions were applied based on the age of diagnosis for CHD or type of ML algorithm used in the study. Studies with an unclear description of reference standard and studies which did not explicitly state the type of cardiac defect were excluded. In studies where both congenital and acquired defects were included, data were extracted only for CHD.

### Data Extraction

Two authors (ZH and UJ) independently extracted information in a pre-formed data extraction sheet. Data obtained included information about the study (first author, year of publication, journal, study title, country, income region of the country according to the World Bank (Organization, 2017), aim of the study, study design, study setting, sample size (including size of training and test set) and method of population selection), the patients (age range, type of CHD), the ML algorithm used (refer to Table 1 (J, 2019), the reference standard, the results (sensitivity, specificity, and area under the curve), validation method of the ML algorithm, and sub-group data if present. Disagreements during the literature selection, data extraction, and risk assessment were resolved by discussion and consensus of the authors. In case of disagreement, a third reviewer (JKD) was involved for final decision.

**TABLE 1:** Categorization and brief description of ML models.

Types of algorithms	Description
Neural networks	Mimics the biological neural network to analyze data
Deep learning	Uses a combination of artificial neural networks in a computationally efficient manner
Ensemble methods	An amalgamation of predictions of multiple weak models used to strengthen overall prediction
Regression algorithms	Maps the relationship between the input and output variable using a measure of error
Regularization methods	It is an extension of regression models but favors simpler models that are generalizable
Clustering methods	An unsupervized machine learning technique that uses the inherent structures in the data to organize the data into groups of maximum commonality
Dimensionality reduction	Similar to clustering but summarizes data using less information
Rule system	Extract rules between variables in the existing dataset to explain observed relationships
Bayesian methods	Explicitly applies Bayes’ theorem for the problem
Decision tree methods	Uses actual values of features in the data to build a model
Instance-based models	Compares new data to the example database (built by the model) using a similarity measure in order to make a prediction
Natural language processing	Converts textual data to a machine readable format

### Risk of Bias Assessment

The risk of bias was assessed by two authors independently using Quality Assessment for Diagnostic Accuracy Studies-2 (QUADAS-2) tool (Whiting et al., 2011). Domains for risk of bias included patient selection, index test, reference standard, and flow and timing with the first three domains also considered in terms of applicability concerns. If one of the questions within the domain was scored at high risk of bias, the domain was scored as high risk.

### Data Analysis

For all included studies, we entered the data provided into Review Manager five software (Review Manager 5.3) (Cochrane, 2008) where the sensitivity, specificity and their 95% confidence intervals (CIs) were presented in the form of forest plots and receiver operating characteristic (ROC) curves. After grouping atleast four studies that used a specific type of ML model (as shown in Table 1), a meta-analysis was performed. This analysis utilized the sensitivity and specificity results from each included study using the *metandi command* for bivariate model in STATA version 16 using (Stata-Corp, College Station, Texas, United States) (StataCorp, 2007) to generate the hierarchical Summary ROC (HSROC) curve.

## Results

**FIGURE 1:**
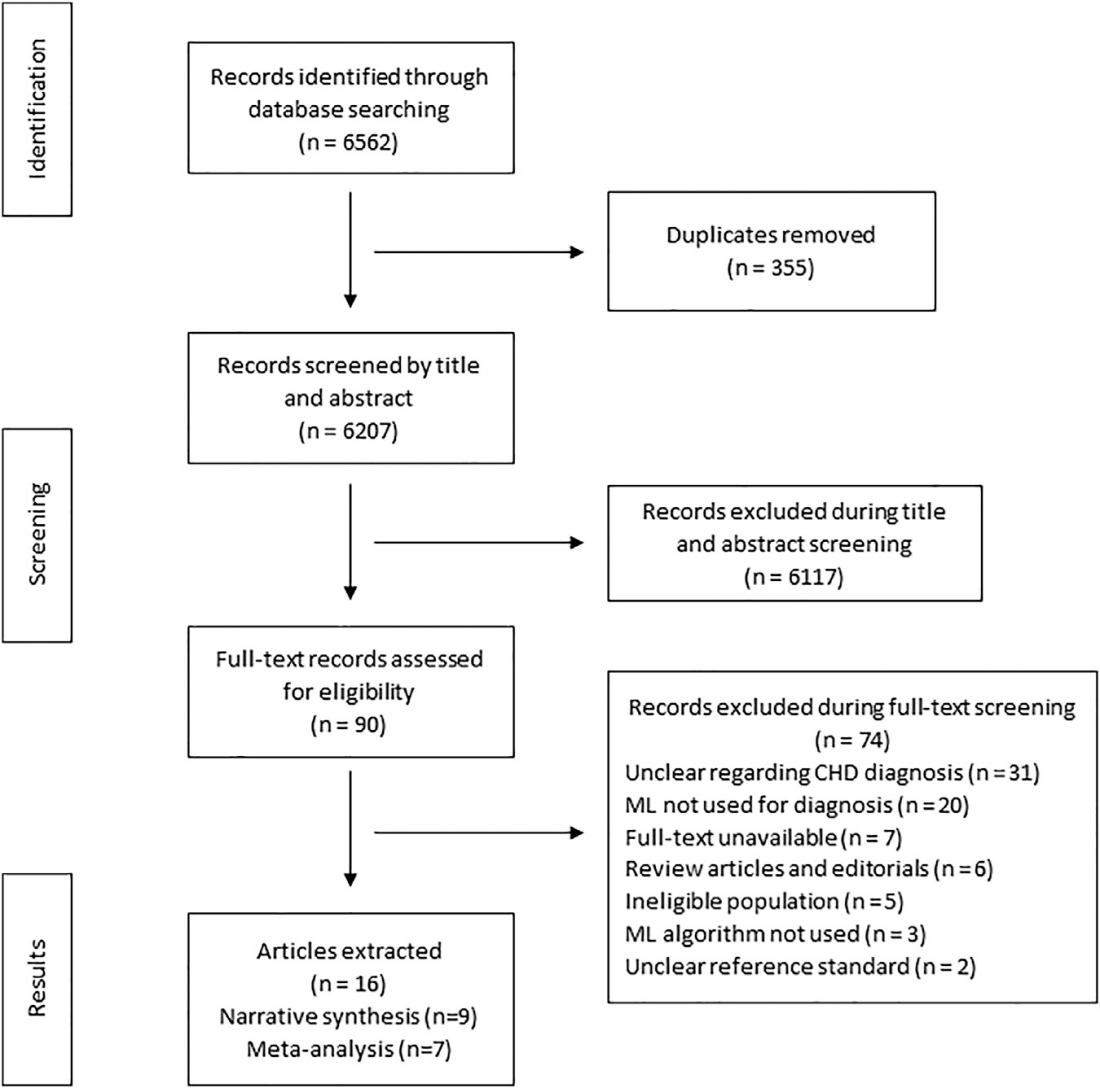
Search flow diagram.

The search strategy identified 6,652 articles from which 90 studies met the eligibility criteria for full text screening. We excluded 74 studies and the remaining 16 studies were included in the review. Out of the 16 included studies; meta-analysis was conducted for seven studies while nine studies were narratively synthesized (refer to Figure 1 for the study flow diagram) (DeGroff et al., 2001; Yang et al., 2002; Bhatikar et al., 2005; Higuchi et al., 2006; De Vos and Blanckenberg, 2007; Ye et al., 2011; Gharehbaghi et al., 2015; Zhang and Pohl, 2015; Gavrovska et al., 2016; Kotb et al., 2016; Sepehri et al., 2016; Karar et al., 2017; Pereira et al., 2017; Meza et al., 2018; Diller et al., 2019a; Bahado-Singh et al., 2020). The characteristics of included studies (*n* = 16) have been outlined in Table 2. Majority of the studies were from HICs (*n* = 10, 62.5%) followed by upper middle income countries (UMICs) (*n* = 4, 25%) and LMICs (*n* = 2, 12.5%). Ten studies (*n* = 62.5%) were case control study designs, five studies (*n* = 31.25%) were cross sectional while one study (*n* = 6.25%) was a cohort design. The sample size of these studies ranged from 22 (Karar et al., 2017) to 824 participants (Kotb et al., 2016). The types of CHDs included Tetralogy of Fallot, Transposition of great arteries, coarctation of aorta, atrial and/or ventricular septal defects, and valvular conditions (stenosis or regurgitation at atrioventricular or semilunar valves). Due to the small number of studies for each of these diagnostic conditions, these were all labeled as an umbrella term of CHD for the purpose of this review. The reference standard for most of the studies was echocardiography (*n* = 9, 56.3%) (DeGroff et al., 2001; Yang et al., 2002; Bhatikar et al., 2005; Higuchi et al., 2006; De Vos and Blanckenberg, 2007; Gharehbaghi et al., 2015; Gavrovska et al., 2016; Kotb et al., 2016; Meza et al., 2018) or the expert clinician (*n* = 7, 43.7%) (Ye et al., 2011; Zhang and Pohl, 2015; Sepehri et al., 2016; Karar et al., 2017; Pereira et al., 2017; Diller et al., 2019b; Bahado-Singh et al., 2020) who made the final diagnosis.

**TABLE 2:** Table of included studies.

Author and year	Country	Income region	Age range	Study design	Input	Index test	Reference standard	Sensitivity (%)	Specificity (%)
Bahado-Singh et al. (2020)	United States	High	Neonates	Case-control	Genetic makeup	DL	Expert	Single genetic marker = 95.51; combination of markers 91.7%	Single genetic marker = 93.8; 3 combination of markers = 87.5%
Bhatikar et al. (2005)	United States	High	Not specified	Case-control	Heart sounds	ANN	Echocardiography	88	83
De Vos and Blanckenberg (2007)	South Africa	Upper-middle	2 months–16 years	Case-control	Heart sounds	ANN	Echocardiography	90	96.46
DeGroff et al. (2001)	United States	High	1 week–15 years	Case-control	Heart sounds	ANN	Echocardiography	100	100
Diller et al. (2019a)	United Kingdom and Germany	High	Adults	Case-control	Images	DL	Expert	NR	NR
Gavrovska et al. (2016)	Serbia	Upper-middle	7–19 years	Cross-sectional	Heart sounds	ANN	Echocardiography	93.1	94.1
Gharehbaghi et al. (2015)	Iran	Upper-middle	2.5–12 years	Cross-sectional	Heart sounds	NN, CSVM	Echocardiography	NN: 84, CSVM: 66.8	NN: 85.7, CSVM: 78.2
Higuchi et al. (2006)	Japan	High	Not specified	Cross-sectional	Heart sounds	ANN	Echocardiography	NR	NR
Karar et al. (2017)	Egypt	Lower-middle	Not specified	Case-control	Heart sounds	Rule-based classification tree	Expert	80	100
Kotb et al. (2016)	Egypt	Lower-middle	1 week–14 years	Cross-sectional	Heart sounds	HMM	Echocardiography	98	89
Meza et al. (2018)	Canada	High	Neonates	Cohort	Images	Cluster analysis	Echocardiography	NR	NR
Pereira et al. (2017)	United States	High	1–7 days	Case-control	Images	SVM	Expert	NR	NR
Sepehri et al. (2016)	Iran	Upper-middle	1–18 years	Cross-sectional	Heart sounds	NN	Expert	87.29	87.89
Yang et al. (2002)	Japan	High	12–56 years	Case-control	ECG	ANN	Echocardiography	91.4	91.7
Ye et al. (2011)	United States	High		Case-control	Images	Non-linear SVM	Expert	95.45	83.33
Zhang and Pohl (2015)	United States	High	Not specified	Case-control	Images	LR	Expert	NR	NR

Notes: ANN: artificial neural network; CSVM: conventional support vector machine DL: deep learning; ECG: electrocardiogram; HMM: hidden markov model LR: logistic regression; MLP: multilayer perceptron; NN: neural network; SVM: support vector machine.

### Methodological Quality of Included Studies

**FIGURE 2:**
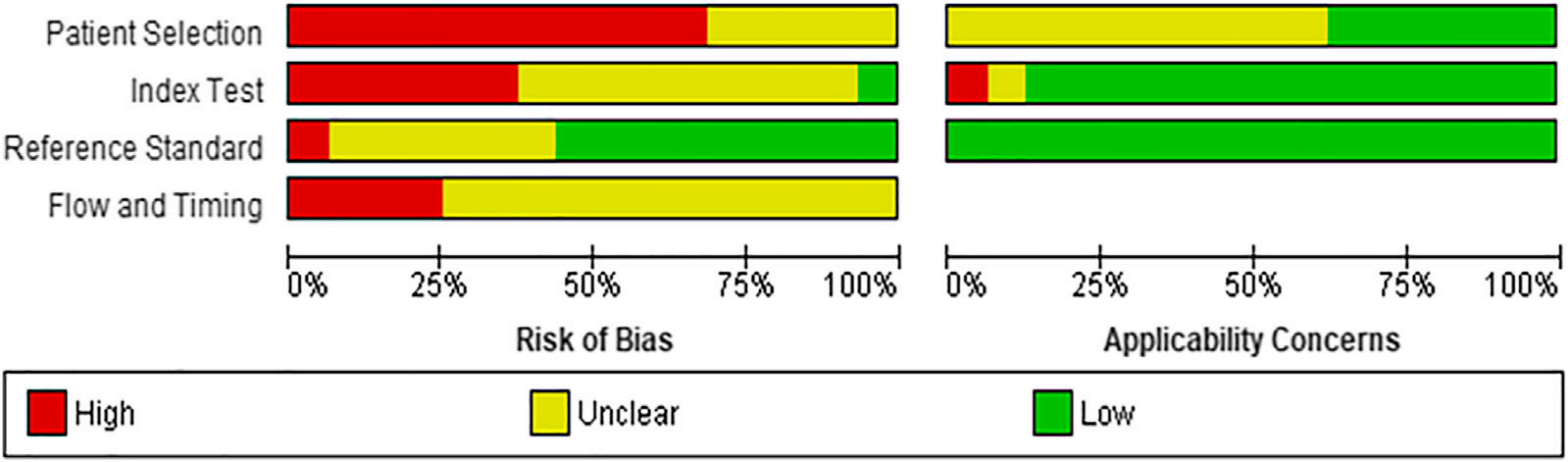
Risk of bias assessment and applicability concerns for included studies.

Risk of Bias assessment reported that eleven studies (69%) (DeGroff et al., 2001; Yang et al., 2002; Bhatikar et al., 2005; De Vos and Blanckenberg, 2007; Ye et al., 2011; Zhang and Pohl, 2015; Karar et al., 2017; Pereira et al., 2017; Meza et al., 2018; Diller et al., 2019a; Bahado-Singh et al., 2020) had high risk of patient selection bias due to the study design (case control) while the remaining five studies (31%) (Higuchi et al., 2006; Gharehbaghi et al., 2015; Gavrovska et al., 2016; Kotb et al., 2016; Sepehri et al., 2016) were unclear risk. The index test interpretation bias was unclear in nine studies (56%) (DeGroff et al., 2001; Yang et al., 2002; Bhatikar et al., 2005; Higuchi et al., 2006; De Vos and Blanckenberg, 2007; Gharehbaghi et al., 2015; Sepehri et al., 2016; Pereira et al., 2017; Diller et al., 2019a), high in six studies (37%) (Ye et al., 2011; Zhang and Pohl, 2015; Gavrovska et al., 2016; Karar et al., 2017; Meza et al., 2018; Bahado-Singh et al., 2020) and low in only one study (7%) (Kotb et al., 2016). The main contributor to the unclear risk was the unavailability of information regarding theblinding status in these studies. Majority of the studies (*n* = 10, 62%) had low risk of reporting bias (DeGroff et al., 2001; Yang et al., 2002; De Vos and Blanckenberg, 2007; Zhang and Pohl, 2015; Gavrovska et al., 2016; Sepehri et al., 2016; Karar et al., 2017; Pereira et al., 2017; Meza et al., 2018; Diller et al., 2019a) while five reported unclear risk (31%) (Bhatikar et al., 2005; Higuchi et al., 2006; Gharehbaghi et al., 2015; Kotb et al., 2016; Bahado-Singh et al., 2020) and one reported high risk (7%) (Ye et al., 2011). The reference standard that was used mainly included expert opinion along with gold standard imaging modalities such as echocardiography, thus reducing the likelihood of bias. On the flow and timing domain, most of the studies (*n* = 12, 75%) (Yang et al., 2002; Higuchi et al., 2006; Ye et al., 2011; Gharehbaghi et al., 2015; Zhang and Pohl, 2015; Gavrovska et al., 2016; Sepehri et al., 2016; Karar et al., 2017; Pereira et al., 2017; Meza et al., 2018; Diller et al., 2019a; Bahado-Singh et al., 2020) were unclear risk of bias as the interval between the index test and reference standard could not be ascertained while four studies (25%) (DeGroff et al., 2001; Bhatikar et al., 2005; De Vos and Blanckenberg, 2007; Kotb et al., 2016) were high risk of bias as they either did not include all patients in the analysis or all participants did not receive the same reference standard. Details of risk of bias and applicability concerns have been highlighted in Figure 2.

No studies had concerns about applicability in the reference standard domain while majority of the studies (*n* = 14, 88%) had low concern on the index text. However, ten studies (62%) had unclear concern on patient applicability.

### Outcome of Interest

The 16 studies included in this review had 1,217 participants where ML models were used to diagnose CHD. Five studies (31%) did not report sensitivity and/or specificity as the performance metric for the ML algorithm. (Higuchi et al., 2006; Zhang and Pohl, 2015; Pereira et al., 2017; Meza et al., 2018; Diller et al., 2019a). The models in these studies included neural networks (*n* = 8), ensemble methods (*n* = 3), deep learning (*n* = 2) as well as other techniques such as rule based classifications and clustering techniques for unsupervized learning.

**FIGURE 3:**
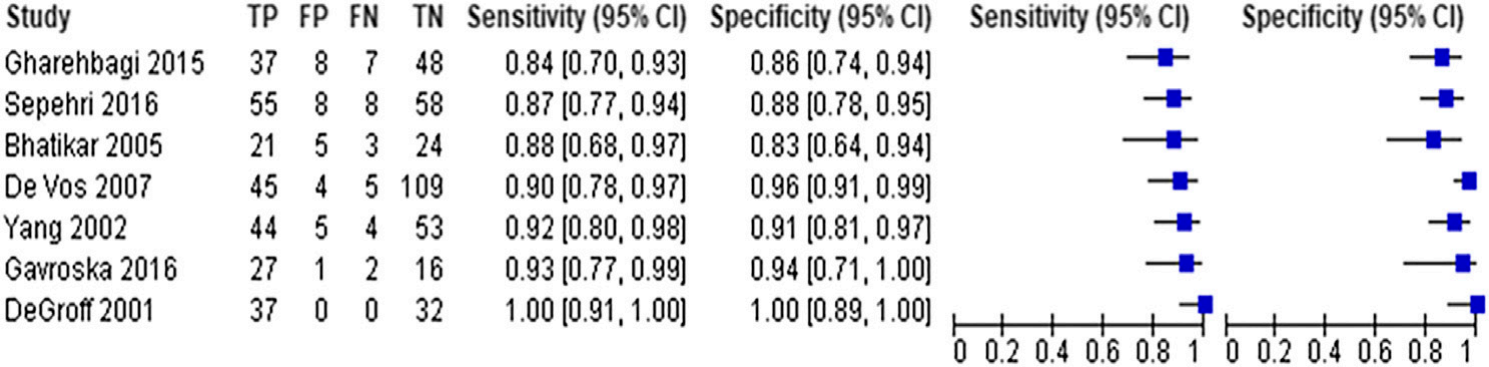
Forest plot for neural network models (arranged by increasing order of sensitivity).

**FIGURE 4:**
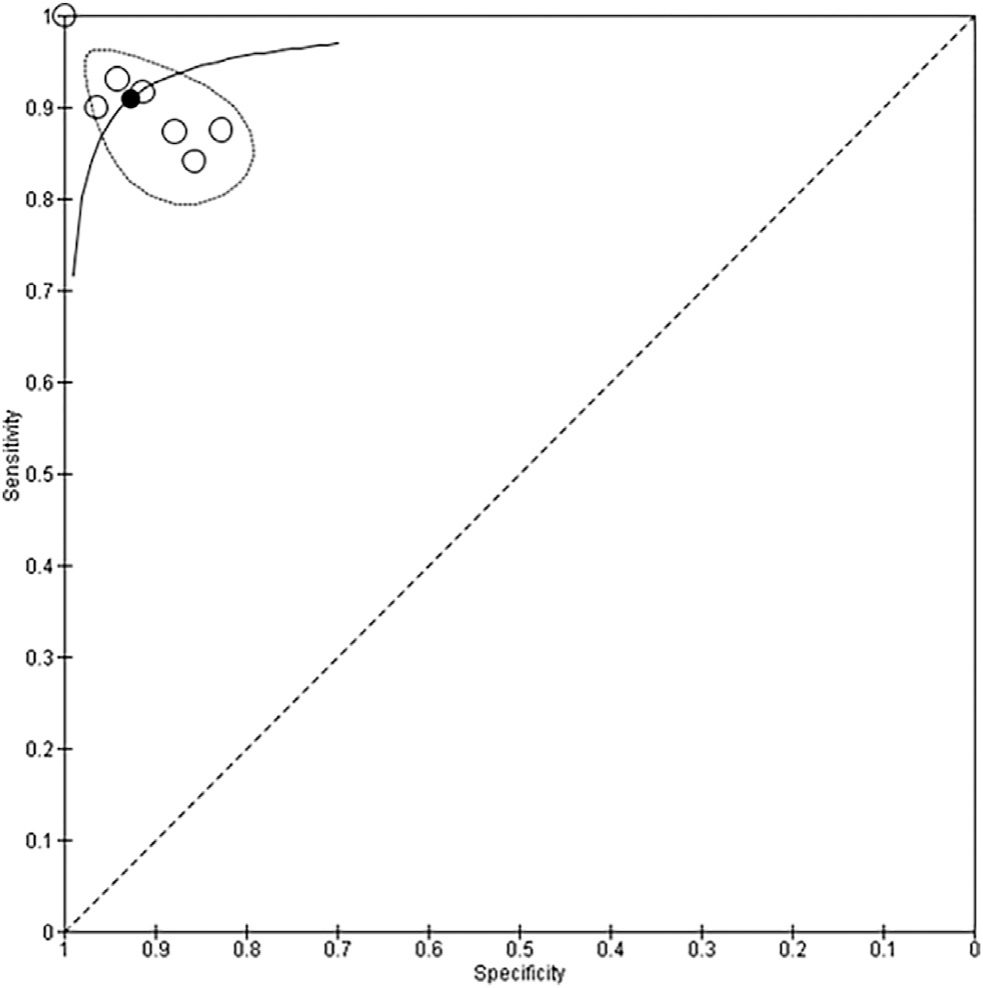
Summary receiver operating curve for use of neural network models to detect CHD.

Seven studies with 666 participants utilized neural networks as the “index text” for detection of CHD (DeGroff et al., 2001; Yang et al., 2002; Bhatikar et al., 2005; De Vos and Blanckenberg, 2007; Gharehbaghi et al., 2015; Gavrovska et al., 2016; Sepehri et al., 2016). Higuchi et al. also used neural networks as the index test but did not report sensitivity and specificity and hence were not included in the analysis (Higuchi et al., 2006). Refer to Figures 3, 4 for the forest plot and ROC curve respectively. Most studies used heart sounds as the input data except for one where electrocardiogram (Yang et al., 2002) was used. The sensitivity of these studies ranged from 84% (Gharehbaghi et al., 2015) to 100% (DeGroff et al., 2001) while the specificity range was from 83% (Bhatikar et al., 2005) to 100% (DeGroff et al., 2001). The overall sensitivity of the neural networks to detect CHD was 90.9% (95% CI 85.2–94.5%) while the overall specificity was 92.7% (95% CI 86.4%–96.2%).

Three studies with 548 participants used the ensemble methods for detection of CHD (Ye et al., 2011; Gharehbaghi et al., 2015; Kotb et al., 2016). However, there was wide variation in the sensitivity (66–100%) and specificity (78–100%) of these studies. One study that used ensemble ML methods did not report these metrics (Pereira et al., 2017) and hence due to an insufficient number of studies, a meta-analysis could not be performed.

One study utilized deep learning methods to detect differences in genetic makeup using newborn blood DNA for diagnosis of CHD (coarctation of aorta) with a sensitivity and specificity of >90% on 40 participants (Bahado-Singh et al., 2020). Deep learning was also utilized by Diller et al. on echocardiographic images but did not report sensitivity and specificity (Diller et al., 2019a). Another study utilized rule based classification tree on heart sounds data of 22 participants with a sensitivity of 80% and specificity of 100% to detect ventricular septal defects (Karar et al., 2017). Regression technique (Zhang and Pohl, 2015), hidden markov models (Kotb et al., 2016) and clustering techniques for unsupervized learning (Meza et al., 2018) were among the other ML techniques utilized but did not report the metrics of interest. Due to the small number of studies that used the ML methods specified and/or lack of information regarding performance metrics, a meta-analysis could not be performed.

## Discussion

To the best of our knowledge, this meta-analysis is the first systematic attempt to assess the diagnostic accuracy of ML models in diagnosing CHD. It was noted that cardiac auscultation and imaging techniques were the main input data sources to the models while neural networks were most commonly used for analysis. This ML technique which was primarily based on heart sounds acquired through a digital stethoscope had a high sensitivity and specificity (>90%) for diagnosis of CHD as compared to expert diagnosis (used as a reference standard). Limited number of studies were available for the other methods such as ensemble method, deep learning, and unsupervized learning, thus precluding a meta-analysis.

In recent years, ML has found several potential applications as decision support in the field of cardiovascular health, with several studies investigating its role in assessment of chamber quantification and cardiac function on imaging (Gandhi et al., 2018), categorization of complex cardiac disease and predicting its prognosis (Diller et al., 2019b). A virtual clinical trial using signal processing techniques and classification algorithms on heart sound to diagnose pediatric CHD showed a sensitivity, specificity, and accuracy of 93, 81, and 88%, respectively (Thompson et al., 2019). Although the performance metrics of ML models to diagnose CHD in isolated studies is promising, a pooled analysis is required to synthesize the evidence regarding the accuracy of new techniques in a systematic manner so that a case for incorporation into clinical practice can be made. The present study reports that one such type of ML model (i.e. neural networks) has a high accuracy to detect CHD using a digital stethoscope without the need of a human interpreter.

Even though the benefits of ML models have been shown in research settings, there is a significant lag between translation of ML models into real world clinical settings. The “productization” of AI technology poses several challenges including large amounts of generalizable datasets, ensuring compliance with regulatory bodies and developing frameworks for integration of these into existing clinical workflows (He et al., 2019). Implementation of AI-based diagnostic tools can have important implications for providing healthcare in resource-limited settings, where existing medical infrastructure (i.e. echocardiography machines) and highly trained skilled providers to obtain and/or interpret the data is inadequate. This meta-analysis provides evidence toward use of a low cost existing tools such as a digital stethoscope that requires minimal operator expertize, and which when coupled with a ML model could have high accuracy as a screening tool to detect CHD in low resource settings.

A recent report by the United States Agency for International Development (USAID) illustrates several examples including the use of a clinical decision support system that can help increase access and quality of care for complex diseases in LMICs (USAID, 2019). Despite this potential implication, published literature on use of ML in healthcare in these regions is lacking. This finding is substantiated by our review where only two (2.5%) studies were published from LMICs in spite of the high burden of CHD in these regions.

In order to increase the clinical applicability of future studies utilizing ML methods for diagnosis, standardization of reporting and performance metrics need to be followed. Adequate descriptions of the study design and flow, important demographic characteristics of patients, data acquisition methods, index test, reference test, standard performance metrics, and thresholds should be provided (Collins et al., 2015). The quality assessment performed in this review highlights the lack of methodological rigor in studies reporting the use of ML in healthcare.

This is the first meta-analysis to present the diagnostic accuracy of ML algorithms for CHD compared to clinical experts or echocardiography, thus highlighting the use of advanced data analytics techniques to improve care especially in regions where highly trained professionals needed for diagnosis of complex disease are limited. However, this study has several limitations. The number of studies eligible for this review were small, thus limiting the ability to perform meta-analyses for only 1 ML method. The heterogeneity of the diagnostic modalities used to train the ML models and the heterogeneity of the CHD diagnoses (critical, major and minor disease) included between the studies is a major limitation. The methodological quality of the studies as assessed by the QUADAS-2 tool was unclear or high for most of the studies. We only included articles published in English language thus leading to a publication bias.

This study highlights the potential of ML models such as neural networks as an accurate decision support tool in diagnosing CHD. However, due to the limited number of studies with high risk of bias, future work would require studies with methodological rigor in assessing the role of advanced AI techniques in detecting CHD accurately.

## Data Availability

The original contributions presented in the study are included in the article/Supplementary Material, further inquiries can be directed to the corresponding author.
